# Evolutionary history of tall fescue morphotypes inferred from molecular phylogenetics of the *Lolium*-*Festuca *species complex

**DOI:** 10.1186/1471-2148-10-303

**Published:** 2010-10-12

**Authors:** Melanie L Hand, Noel OI Cogan, Alan V Stewart, John W Forster

**Affiliations:** 1 Department of Primary Industries, Biosciences Research Division, Victorian AgriBiosciences Centre, 1 Park Drive, La Trobe University Research and Development Park, Bundoora, Victoria 3083, Australia; 2 Molecular Plant Breeding and Dairy Futures Cooperative Research Centres, Australia; 3 La Trobe University, Bundoora, Victoria 3086, Australia; 4 PGG Wrightson Seeds, P.O. Box 175, Lincoln 7640, Canterbury, New Zealand

## Abstract

**Background:**

The agriculturally important pasture grass tall fescue (*Festuca arundinacea *Schreb. syn. *Lolium arundinaceum *(Schreb.) Darbysh.) is an outbreeding allohexaploid, that may be more accurately described as a species complex consisting of three major (Continental, Mediterranean and rhizomatous) morphotypes. Observation of hybrid infertility in some crossing combinations between morphotypes suggests the possibility of independent origins from different diploid progenitors. This study aims to clarify the evolutionary relationships between each tall fescue morphotype through phylogenetic analysis using two low-copy nuclear genes (encoding plastid acetyl-CoA carboxylase [*Acc1*] and *centroradialis *[*CEN*]), the nuclear ribosomal DNA internal transcribed spacer (rDNA ITS) and the chloroplast DNA (cpDNA) genome-located *matK *gene. Other taxa within the closely related *Lolium*-*Festuca *species complex were also included in the study, to increase understanding of evolutionary processes in a taxonomic group characterised by multiple inter-specific hybridisation events.

**Results:**

Putative homoeologous sequences from both nuclear genes were obtained from each polyploid species and compared to counterparts from 15 diploid taxa. Phylogenetic reconstruction confirmed *F. pratensis *and *F. arundinacea *var. *glaucescens *as probable progenitors to Continental tall fescue, and these species are also likely to be ancestral to the rhizomatous morphotype. However, these two morphotypes are sufficiently distinct to be located in separate clades based on the ITS-derived data set. All four of the generated data sets suggest independent evolution of the Mediterranean and Continental morphotypes, with minimal affinity between cognate sequence haplotypes. No obvious candidate progenitor species for Mediterranean tall fescues were identified, and only two putative sub-genome-specific haplotypes were identified for this morphotype.

**Conclusions:**

This study describes the first phylogenetic analysis of the *Festuca *genus to include representatives of each tall fescue morphotype, and to use low copy nuclear gene-derived sequences to identify putative progenitors of the polyploid species. The demonstration of distinct tall fescue lineages has implications for both taxonomy and molecular breeding strategies, and may facilitate the generation of morphotype and/or sub-genome-specific molecular markers.

## Background

The *Festuca *genus is the largest within the Loliinae subtribe of the Poaceae family, and contains over 500 species of temperate grasses [1]. Species of *Festuca *vary in morphology, with studies of leaf anatomy and phylogeny based on sequence of the internal transcribed spacer (ITS) region of ribosomal DNA (rDNA) consistently defining two major evolutionary lineages, of broad and fine-leaved species [1-6]. The genus also varies substantially in ploidy levels, from diploid (2n = 2x = 14) to dodecaploid (2n = 12x = 84), the vast majority of species being allopolyploid [7,8]. One of the most agriculturally important *Festuca *species is tall fescue (*Festuca arundinacea *Schreb.); a broad-leaved outbreeding allohexaploid grass that is cultivated for pasture production throughout the temperate world. Within the *Festuca *genus, tall fescue has been recognised as belonging, along with other mostly polyploidy species, to the *Schedonorus *sub-genus [2]. This taxonomic classification has been the subject of some controversy, as *Schedonorus *species share a close relationship with *Lolium*, the relatively less populated genus of ryegrasses and allied species, which contains ten recognised diploid taxa [9-11]. The monophyly of *Schedonorus *and *Lolium *has led to proposals of reclassification, such that the *Schedonorus *sub-genus is aligned within *Lolium *and tall fescue is hence renamed *Lolium arundinaceum *(Schreb.) Darbysh [12]. The *Lolium *and *Festuca *genera undoubtedly represent a closely allied complex of related and partially interfertile species. In this study, however, due to comparisons of tall fescue and other broad-leaved species with taxa which remain classified as part of *Festuca*, the nomenclature and sub-generic classification of Clayton and Renvoize [2] is retained.

The *Schedonorus *sub-genus is itself a complex species group with considerable ploidy variation resulting from multiple combinatorial hybridisation events. Within this sub-genus, hexaploid tall fescue is a member of a polyploid series that consists of a tetraploid (*F. arundinacea *var. *glaucescens *Boiss. = *F. arundinacea *subsp. *fenas *(Lag.) Arcang.), octoploid (*F. arundinacea *subsp. *atlantigena *(St. Yves) Auquier) and a decaploid (*F. arundinacea *subsp. *letourneuxiana *St. Yves.). Other evolutionary important *Schedonorus *species include diploid meadow fescue (*F. pratensis *Huds.), *F. pratensis *subsp. *apennina *(De Not.) Hegi (tetraploid), *F. mairei *St. Yves (tetraploid) and the hexaploid *F. gigantea *(L.) Vill. The complex evolutionary relationships between these species have to date been studied through the generation of hybrids [13-17], cytological analysis [18], *in situ *hybridisation [19-22], molecular genetic marker variation [3,23,24] and comparison of chloroplast and rDNA ITS nucleotide sequence [1,4-6].

Further complications arise due to variation within hexaploid tall fescue itself, which may be more accurately described as a species complex. Three major forms of tall fescue have been recognised (Continental, Mediterranean and rhizomatous) that differ in terms of agronomically significant morphological and physiological attributes. These distinct forms are denoted in this study as morphotypes. The summer-active Continental type that predominates in Northern Europe has contributed the majority of temperate cultivated germplasm, and has been the subject of most published tall fescue studies. The Mediterranean type endemic to Northern Africa, parts of Italy and the Middle East displays incomplete summer dormancy and greater winter growth, but lacks winter hardiness as compared to the Continental type [25,26]. The two morphotypes also appear to harbor distinctly different symbiotic fungal endophytes of the *Epichloë *type [27,28].

The third, rhizomatous, morphotype predominates in parts of northern Portugal and Galicia in Spain [29,30] and is distinguished by the presence of both longer and more prevalent rhizomes than those seen in Continental and Mediterranean germplasm, as well as some other distinct taxonomic traits [30,31]. Rhizomatous tall fescue has hence become the target of turf breeding programs due to its superior spreading ability, firstly in New Zealand [32-35] and later in Europe and the USA [36].

The observed differences between the three types would not necessarily be of taxonomic significance, except that F_1 _hybrids between morphotypes, despite being highly vigorous individuals, may display infertility. This property has been reported for crosses between Continental and Mediterranean morphotypes, which display irregular meiotic pairing resulting from extensive multivalent formation [37-43]. Similarly, hybrids between rhizomatous and Mediterranean plants are highly desynaptic, forming numerous univalents at meiosis [43], and hence sterile [34,36]. The sterility effects have in part been explained in terms of genetic control of a diploid-like chromosome pairing mechanism in hexaploid tall fescue [44-46]. This mechanism, which may be of broad occurrence in the Poaceae family, does not operate at the haploid level (and is hence termed haplo-insufficient) [44,45]. It is also possible that failure of meiotic pairing is due to structural or genomic differences between chromosomes [47]. Many allopolyploid species have arisen more than once from different progenitor ancestral species [48] and it is probable that hexaploid tall fescue follows this pattern, with separate origin events in different regions [30,49].

Attempts to determine the genomic constitution of tall fescue have to date solely focused on the Continental morphotype. Meadow fescue (*F. pratensis*) has previously been identified as the contemporary taxon most closely related to one of the diploid progenitor sub-genome (P) donors, based on chromosome structure and pairing [13,17] studies. The two remaining sub-genomes (G_1 _and G_2_) have been attributed to the tetraploid *F. arundinacea *var. *glaucescens*, based on genomic *in situ *hybridisation (GISH) and other molecular genetic techniques [19,20,23]. The genomic constitution of Continental tall fescue has therefore been designated PPG_1_G_1_G_2_G_2_.

Although multiple studies of *Festuca *genus phylogeny have been performed [1,3-6,24], none as yet have included multiple tall fescue morphotypes. Furthermore, the methods employed were limited in capacity to identify each putative progenitor of an allopolyploid species. Genes present in single copy or low copy-number within the nuclear genome have become increasingly popular for studying plant phylogenies [50] and have been used to determine the hybrid origins of polyploid species [51-54]. As compared to the sequences that have been traditionally used for molecular evolutionary studies, nuclear genes provide sequence evolution rates elevated in comparison to chloroplast DNA (cpDNA) and rDNA [55,56], are biparentally inherited (in contrast to cpDNA) and are less frequently subjected to concerted evolution than rDNA [57]. This study aims to compare the sequence of two protein-coding genes (*Acc1 *[encoding plastid acetyl-CoA carboxylase] and *CEN *[the floral developmental identity-determining gene *centroradialis*, also known as *terminal flower 1*]) obtained from all three tall fescue morphotypes, along with some other species of section *Schedonorus*, and taxa previously identified as putative diploid progenitors. The *Acc1 *gene is present as a single copy in *Triticum *(wheat) species on the homoeologous group 2 chromosomes [58] and has been used to achieve high phylogenetic resolution and identify the hybrid origins of a number of polyploid species [59-61]. The *CEN *gene has been previously isolated and characterised in perennial ryegrass (*Lolium perenne *L.) and although Southern hybridisation studies revealed two gene copies in this species, only one was detected and isolated from a genomic library [62]. The *LpCEN *gene has been previously mapped to perennial ryegrass linkage group 5 [63]. Given the large extent of macrosynteny between the genomes of the Triticeae cereals and both *Lolium *and *Festuca *species, the allohexaploid tall fescue *Acc1 *and *CEN *orthologues are likely to be located on homoeologous linkage groups 2 and 5, respectively [64-66]. The value of the *CEN *gene for molecular phylogenetics studies in Poaceae species has been evaluated here for the first time. This study is hence aimed at increasing knowledge of evolutionary relationships between each tall fescue morphotype, and other species of the *Schedonorus *sub-genus through the use of three classes of gene sequence: the nuclear rDNA ITS region, the two low-copy nuclear genes, and the cpDNA *maturase K *(*matK*) gene, which has been adopted as an international standard for DNA 'barcoding' [67] and provides evidence for maternal progenitor identity.

## Methods

### Orthology assessment

Exonic regions of the wheat *Acc1 *(GenBank accession EU660902) and the perennial ryegrass *CEN *(GenBank accession AF316419) nuclear genes were used as subject queries in BLASTN analysis [68] of the *Brachypodium distachyon *genome sequence 8X release [69], to determine copy number in the model species, and hence probable abundance of predicted orthologues (defined at a threshold level of E < 10^-50^) in *Festuca *species.

### Sampling and DNA Extraction

Sampling of *Lolium*/*Festuca *taxa was designed to include the three tall fescue morphotypes along with diploid and polyploid species that are thought to share common sub-genomes (Table 1). A sample from the related grass species crested dog's-tail (*Cynosurus cristatus *L.) was chosen as an out-group for all datasets. Seed of each tall fescue morphotype was supplied by PGG Wrightson Seeds, apart from the Continental cultivar KY31, which was obtained from the Royal Barenbrug Group. The remainder of the samples used were sourced from the Genetic Resources Unit, Institute for Biological, Environmental and Rural Studies (IBERS), Aberystwyth, Wales. Genomic DNA was extracted from freeze-dried leaf tissue from one individual per cultivar or accession, using the DNeasy Plant Mini Kit (Qiagen)

**Table 1:** List of samples included in the study and details of ploidy levels and origin

Taxon	Ploidy	Origin	Sub-genus	IBERS Accession Identifier	Number of distinct haplotypes	
					*Acc1*	*CEN*
*Festuca arundinacea *Schreb.	6x					
Jesup		Continental cultivar	*Schedonorus*	N/A	3	3
Quantum		Continental cultivar	*Schedonorus*	N/A	3	3
KY31		Continental cultivar	*Schedonorus*	N/A	3	3
Resolute		Mediterranean cultivar	*Schedonorus*	N/A	2	2
PG4012		Mediterranean breeding line	*Schedonorus*	N/A	2	2
Torpedo II		Rhizomatous cultivar	*Schedonorus*	N/A	3	3
CT2093R		Rhizomatous breeding line	*Schedonorus*	N/A	3	3
*Festuca pratensis *Huds.	2x	Tadham Moor (Great Britain)	*Schedonorus*	BF1199	1	1
*Festuca pratensis *subsp. *apennina *(De Not.) Hegi	4x	Le Moleson (Switzerland)	*Schedonorus*	BF954	2	2
*Festuca arundinacea *var. *glaucescens *Boiss	4x	Aoiz (Spain)	*Schedonorus*	BN581	2	2
*Festuca mairei *St. Yves	4x	Marrakesh (Morocco)	*Schedonorus*	BS3065	2	2
*Festuca gigantea *(L.) Vill.	6x	Unspecified	*Schedonorus*	BS5001	2	3
*Festuca arundinacea *subsp. *atlantigena *(St. Yves) Auquier	8x	Unspecified	*Schedonorus*	BN865	3	2
*Festuca arundinacea *subsp. *letourneuxiana *St. Yves	10x	Agadir Gouj (Morocco)	*Schedonorus*	BN812	4	4
*Festuca drymeja *Mert. & Koch	2x	(Hungary)	*Drymanthele*	BS3675	1	1
*Festuca altissima *All.	2x	Koskeg (Hungary)	*Drymanthele*	BS4384	1	1
*Festuca lasto*	2x	Los Barrios (Spain)	*Drymanthele*	BS3748	1	1
*Festuca scariosa *(Lag) Asch. & Graebn.	2x	Unspecified	*Scariosae*	BS3278	1	1
*Festuca pallens *Host.	2x	Gyulakeszi (Hungary)	*Festuca*	BL2811	1	1
*Festuca circummediterranea *Patzke	2x	Colle Sanson (Italy)	*Festuca*	BL2787	1	1
*Festuca rupicaprina *(Hackel) A. Kerner	2x	Unspecified	*Festuca*	BL2767	1	1
*Festuca tatrae *(Czako) Degen	2x	Unspecified	*Festuca*	BL2758	1	1
*Festuca ovina *L.	2x	Ponterwyd (Great Britain)	*Festuca*	BL2643	1	1
*Festuca valesiaca *Schleicher ex Gaudin	2x	Pregradnaya (Russia)	*Festuca*	BL2506	1	1
*Lolium perenne *L.	2x	Cultivar 'Aurora'	-	N/A	1	1
*Lolium multiflorum *Lam.	2x	Cultivar 'Andrea'	-	N/A	1	1
*Lolium temulentum *L.	2x		-	BA13157	1	1
*Cynosurus cristatus *L.	2x	Ponterwyd (Great Britain)	-	BG527	1	1

N/A = not applicable

### Ploidy Analysis

Ploidy levels for each sample were confirmed using flow cytometric measurements obtained using a Partec Ploidy Analyser instrument. For each cultivar or accession, the single individual from which genomic DNA was extracted was also used for DNA content measurement. Nuclei were extracted from fresh leaf tissue and stained using CyStain UV precise P (Partec) according to the manufacturer's instructions. Prepared samples were immediately analysed using ultraviolet (UV) light excitation, and duplicate readings were obtained for each sample. A total of two separately prepared samples were measured for each individual tall fescue plant. The genome size for each species was measured relative to *F. pratensis *(as a confirmed diploid standard) and then compared to previously published relative genome sizes [8,18,70,71].

### PCR Amplification

PCR reactions were established in total volumes of 20 μl, containing 1 × Immolase PCR buffer, 1.5 mM MgCl_2_, 200 μM dNTPs, 0.25 μM each primer (listed in Table 2), 0.4 units Immolase DNA polymerase (Bioline) and 10 ng template genomic DNA. For the *Acc1 *and rDNA ITS regions, PCR programs were as previously described (references listed in Table 2). The *CEN *gene fragment was amplified using a touchdown PCR program consisting of 95°C for 15 minutes, 10 cycles of 95°C for 30 seconds, 65°C for 30 seconds decreasing 1°C per cycle, 72°C for 2 minutes, followed by 25 cycles of 95°C for 30 seconds, 55°C for 30 seconds to 72°C for 2 minutes and a final extension of 72°C for 7 minutes. To amplify the *matK *gene region, the same touchdown protocol was used with an altered initial annealing temperature of 60°C.

**Table 2:** Details of primer pairs used to amplify the rDNA ITS region and the *matK*, *Acc1 *and *CEN *genes

DNA region	Primer name	Primer sequence 5' - 3'	Reference
*Acc1*	Acc1f1	GTTCCTGGCTCCCCAATATTTATC	[59]
	Acc1r1	TTCAAGAGATCAACTGTGTAATCA	[59]
*CEN*	CENf1	TAAGCAGCCCAAGCCCTTCAAAG	This paper
	CENr3	CGAGGAAGTAATGTAGAAGAGGAGC	This paper
ITS	ITSL	TCGTAACAAGGTTTCCGTAGGTG	[72]
	ITS4	TCCTCCGCTTATTGATATGC	[72]
*matK*	S5-1F	ACCCTGTTCTGACCATATTG	[94]
	trnK-2R	AACTAGTCGGATGGAGTAG	[95]

### Cloning and Sequencing

All *Acc1 *and *CEN *amplification products were sub-cloned into the pCR^®^4-TOPO^® ^vector using the TOPO^® ^TA Cloning^® ^Kit for Sequencing (Invitrogen) and were transformed into TOPO10 chemically competent *E. coli *cells following manufacturer's instructions. For each plant accession-gene combination, 12 colonies were picked for each predicted diploid sub-genome, and the recombinant plasmid DNA was amplified using the TempliPhi DNA Sequencing Template Amplification Kit (GE Healthcare). The amplified template was diluted with the addition of 56 μl of ddH_2_O and 2 μl used as template for sequencing with T7 and T3 primers. Each sequencing reaction contained 5 μl total volume and contained 0.16 μM primer, 0.125 μl BigDye^® ^Terminator v3.1 (Applied Biosystems), 0.875 × BigDye^® ^Sequencing Buffer (Applied Biosystems) and was subjected to cycling conditions as described in the BigDye^® ^v.3.1 protocol. The extension products were purified with ethanol, sodium acetate and EDTA following the BigDye^® ^v3.1 protocol (Applied Biosystems) and electrophoresis was performed on the ABI3730xl automated capillary electrophoresis platform.

The amplified ITS and *matK*-derived amplicons were purified using Exonuclease I and shrimp alkaline phosphatase and a total of 0.5 μl purified PCR product was sequenced in a 10 μl total volume reaction containing the previously listed reagents, with amendment of the volume of BigDye^® ^v.3.1 (0.25 μl). The ITSL, ITS4, ITS2 and ITS3 primers [72] were used to fully sequence the ITS region, and the *matK *gene was sequenced using the amplification primers.

### Sequence alignment and analyses

To separate homoeologous sequence haplotypes derived from *Acc1 *and *CEN*, all sequences obtained from each sample were first aligned using Sequencher 4.7 (Gene Codes). Pairwise distances between all sequences from a given accession were calculated using the maximum composite likelihood nucleotide substitution model implemented in MEGA version 4 [73]. The diploid samples were first analysed to determine a p value threshold that could be used to discriminate between probable allelic (homologous) variation and homoeologous sequence diversity. Each identified group of sequences falling below this threshold were then separately aligned, and consensuses were defined for phylogenetic analysis. These putative homoeologous consensus sequences are hereafter described as haplotypes.

For each gene, all haplotypes were aligned using Sequencher 4.7 (Gene Codes) and edited manually as required. For the directly sequenced ITS and *matK *gene products, ambiguous bases were denoted using the standard IUPAC ambiguity codes, and boundaries of the ITS spacers and the 5.8 S gene were determined as previously described [4]. For *Acc1 *and *CEN*, intron-exon boundary coordinates were estimated according to those previously determined in bread wheat (*Triticum aestivum *L.) [58] and perennial ryegrass [62]. Measures of variation (variable sites, parsimony informative sites and indels) within the aligned contigs were calculated using MEGA version 4 [73] and DnaSP version 4.90.1 [74].

### Phylogenetic reconstruction

Prior to performing phylogenetic analysis, all gaps were coded as binary characters using GapCoder [75]. For each data matrix, parsimony analysis was performed using PAUP* v4.0b10 [76] with heuristic search options, TBR branch swapping and 1000 replicates of random sequence addition. Characters were equally weighted and statistical support was achieved through bootstrapping using 1000 replicates [77].

*C. cristatus *was used as an out-group species for all data sets, and sequences deposited in GenBank from kentucky bluegrass (*Poa pratensis *L.) and *B. distachyon *were used as more distant out-group species for the more conserved rDNA ITS and *matK *sequence datasets (GenBank accessions AY237833, AF303399, AF164402, AM234568). The *B. distachyon Acc1 *and *CEN *genes identified in the orthology assessment were also used as out-group sequences for each respective nuclear gene data set. The Incongruence Length Difference test [78] was used to assess congruence between the nuclear gene data sets and was implemented in PAUP* v4.0b10 through the partition homogeneity test with 1000 replicates.

## Results

### Orthology assessment

The largest exons (numbers eight and ten of *Acc1*, and two and four of *CEN*) were used to search the *Brachypodium distachyon *genome sequence. For both genes, BLASTN analysis retrieved a single sequence with a below-threshold E value. The *Acc1 *orthologue is located on *B. distachyon *chromosome 5, corresponding to the predicted gene bd5g03860, while the *CEN *orthologue is annotated as predicted gene bd4g42400 on chromosome 4.

### Ploidy analysis

For all but one sample tested, the relative genome size did not differ by greater than 0.6-fold when calculated using either the measurements of the Ploidy Analyser or from genome size values previously published (Additional file 1). These samples were therefore deemed to have the predicted ploidy level (Table 1). The *F. rupicaprina *sample differed by over two-fold when comparing the two relative genome size measurements, and was subsequently removed from all further analysis.

### Isolation of homoeologous sequences

For both *Acc1 *and *CEN*, the analysis of pairwise distance between sequences from diploid samples failed to produce a p value of greater than 0.01. Therefore, variation between any two sequences with a p value less than 0.01 was predicted to be the result of allelic variation or sequence error. For the polyploid samples, sequences with a p value of greater than 0.01 were therefore considered likely to be of different sub-genome origin. Each identified group of homoeologous sequences were separately aligned and consensuses were defined for phylogenetic analysis. The number of haplotypes identified for each sample is detailed in Table 1.

### Sequence analysis

#### ITS

The amplified ITS region used for phylogenetic analysis consisted of the gene encoding the 5.8 S ribosomal subunit, as well as the two ITS regions that separate this gene from the 18 S and 26 S ribosomal subunit genes (ITS1 and ITS2 respectively). The amplified region ranged from 595 to 599 bp, length variation being attributable to the ITS1 spacer. Both the 5.8 S and ITS2 regions were the same length for all samples, at 269 and 101 bp respectively. After alignment of all ITS sequences, the total contig length was 610 bp and contained 174 variable sites and 100 parsimony informative sites (Table 3). A total of 25 of the 33 sequenced samples contained at least one ambiguous base. These sequences were deposited in GenBank under the accession numbers HM453173 - HM453199 and the final sequence alignment is included as Additional File 2. For each sample, the generated ITS sequence was compared to accessions from the same species that were previously submitted in GenBank from other studies. This evaluation, along with the comparisons of genome size calculated in this study with prior reports in the literature, was used to confirm the identity of each species.

**Table 3:** Characteristics of each sequenced region

	Gene			
	
	*Acc1*	*CEN*	ITS	*matK*
Total aligned length (bp)	2254	972	610	1545
Exon length (bp) min-max	40-188	41-218	-	-
Average exon length	101	128	-	-
Exon std dev min-max	0-0	0-0	-	-
Intron length (bp) min-max	73-493	82-958	-	-
Average intron length	110	119	-	-
UTR length (bp) min-max	-	42-56	-	-
Average UTR length	-	55	-	-
Exon + intron + (UTR) length (bp) min-max	1525-1946	903-958	-	-
Average exon + intron + (UTR) length (bp)	1572	925	-	-
Variable sites	492	275	174	179
Parsimony informative sites	199	122	100	70
Indels	112	44	22	4
Indel length (bp) min-max	1-413	1-32	1-4	6-9

#### matK

The amplicon generated using the published *matK *primers contained an extra 65 and 288 bp from the 5'- and 3'-boundaries, respectively, of the *matK *gene. However, only the gene itself was used for analysis. These sequences were deposited in GenBank under the accession numbers HM453050 - HM453076 and the final sequence alignment is included as Additional File 3. The *matK *gene was 1542 bp in length for all samples with the exception of *C. cristatus *(1545 bp). The total contig length used for phylogenetic analysis was 1545 bp and contained 179 variable sites, and 70 parsimony informative sites (Table 3).

#### Acc1

For the majority of taxa, the number of haplotypes obtained was equivalent to the number of predicted sub-genomes for each species (Table 1). The exceptions were *F. gigantea*, *F. arundinacea *subsp. *atlantigena*, *F. arundinacea *subsp. *letourneuxiana *and the two Mediterranean morphotype varieties Resolute and PG4012, for which the number of derived haplotypes was one less than the ploidy level. The amplified gene fragment contained 8 exons and 7 introns, corresponding to exons 6 to 13 of the full-length wheat gene. The total length ranged from 1525 to 1946 bp, the major size disparities being due to insertions in the first and second introns. *F. valesiaca *contained a 235 bp insertion within the first intron, while a 390 bp insertion was present within the second intron for one haplotype derived from *F. arundinacea *subsp. *atlantigena*. The 8 exons ranged in size from 40 to 188 bp: however there was no size variation between samples within each exon. The 7 introns ranged in size from 73 to 493 bp, and each intron displayed more variation between haplotypes than the exons. The total aligned length of the contig was 2254 bp which included 492 variable sites, 199 parsimony informative sites and 112 indels (Table 3). These haplotypes were deposited in GenBank under the accession numbers HM453077 - HM453124 and the final sequence alignment is included as Additional File 4.

#### CEN

As for *Acc1*, in the majority of instances the number of haplotypes recovered for a given species was equivalent to the predicted sub-genome number (Table 1). The exceptions were *F. arundinacea *subsp. *letourneuxiana *and the two Mediterranean morphotype tall fescue varieties Resolute and PG4012, for which haplotype number was one less than the ploidy level, and the octoploid *F. arundinacea *subsp. *atlantigena*, from which only two haplotypes were recovered. The amplified gene fragment ranged in size from 903 to 958 bp and contained 4 exons, 3 introns and a region of the 3'-untranslated region (UTR). The 4 exons ranged in size from 41 to 218 bp, also exhibiting no size variation between species and haplotypes. Greater variation was observed between species with respect to intron length, as well as the size of the 3'-UTR which ranged from 42 to 56 bp in size. The total aligned length of the contig was 972 bp, which included 275 variable sites, 122 parsimony informative sites and 44 indels (Table 3). The haplotypes were deposited in GenBank under the accession numbers HM453125 - HM453172 and the final sequence alignment is included as Additional File 5.

### Phylogenetic inference

#### ITS

**Figure 1:**
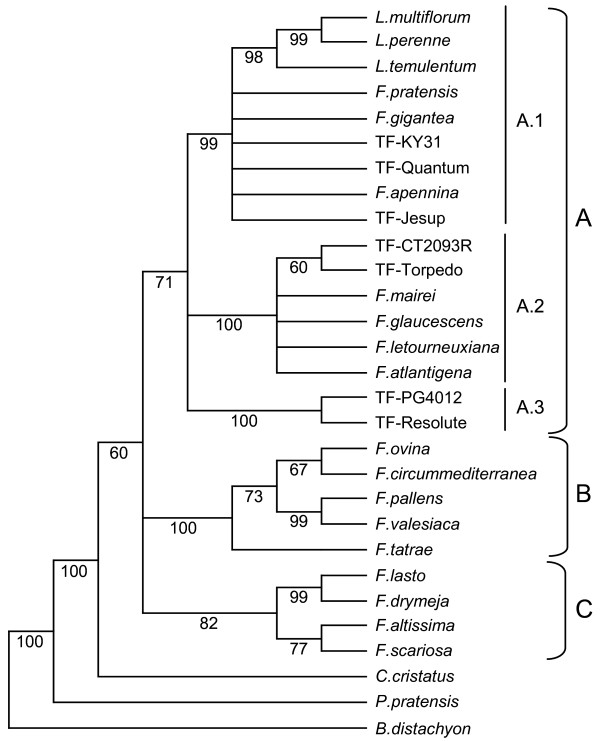
**Strict consensus tree obtained from sequence analysis of the nuclear rDNA ITS region**. Numbers below branches are bootstrap percentages

The parsimony analysis yielded 36 most parsimonious trees (CI = 0.738;RI = 0.828), the strict consensus of which is shown in Figure 1. This tree resolved three major lineages, in which clades A and B represent the *Schedonorus*/*Lolium *and *Festuca *sub-genera respectively, and clade C contains representatives of the *Drymanthele *and *Scariosae *sub-genera. Clade A (71% of bootstrap) is further resolved into three well-supported groups (A.1, A.2 and A.3, at 99, 100 and 100% bootstrap levels) which separate the three tall fescue morphotypes. A.1 contains the resolved diploid *Lolium *representatives and six unresolved samples including the three Continental tall fescue varieties, along with one diploid (*F. pratensis*), one tetraploid (*F. pratensis *subsp. *apennina*) and one other hexaploid (*F. gigantea*). Clade A.2 is composed of the two rhizomatous tall fescue varieties (Torpedo and CT2093R) as well as four unresolved samples (*F. mairei*, *F. arundinacea *var. *glaucescens*, *F. arundinacea *subsp. *letourneuxiana *and *F. arundinacea *subsp. *atlantigena*), while clade A.3 consists solely of the two Mediterranean tall fescue varieties (Resolute and PG4012). The diploid fine-leaved species of the *Festuca *sub-genus form group B (100% of bootstrap), and the third major lineage, C (82% bootstrap), is composed of three *Drymanthele *sub-genus representatives, two which share a sister relationship (*F. lasto *and *F. drymeja*), and the third (*F. altissima*) is related to the one representative of the *Scariosae *sub-genus (*F. scariosa*). *C. cristatus*, *P. pratensis *and *B. distachyon *were out-groups to the entire tree, as anticipated.

#### matK

**Figure 2:**
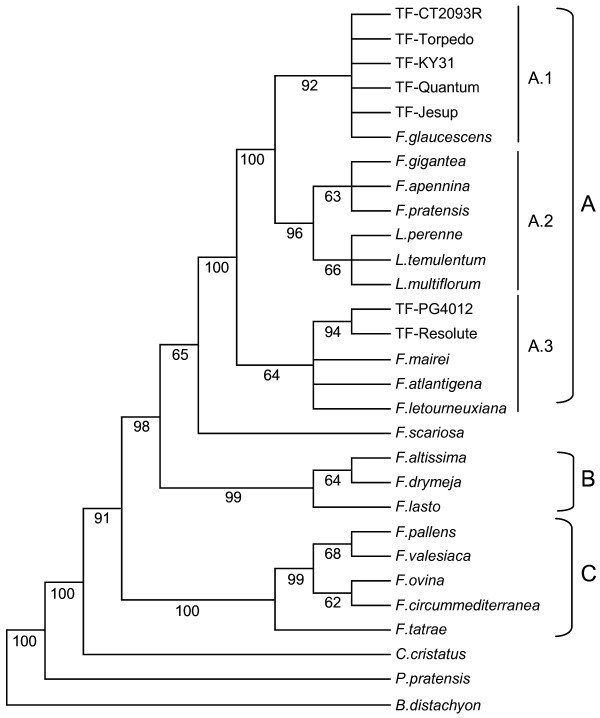
**The single most parsimonious tree obtained from sequence analysis of the cpDNA *matK *gene**. Numbers below branches are bootstrap percentages

A single most parsimonious tree (CI = 0.943, RI = 0.967) resolved into three major clades (A-C) was generated from the heuristic search conducted on the *matK *data matrix (Figure 2). Clade A was further divided into two well-supported sub-clades (A.1 and A.2: 92% and 96% of bootstrap respectively) and a third less-well supported sub-clade A.3 (64% of bootstrap). *F. arundinacea *var. *glaucescens *and the Continental and rhizomatous tall fescue varieties were unresolved in A.1, while A.2 consists of the three unresolved *Lolium *species in sister relationships to the unresolved group containing *F. pratensis*, *F. pratensis *subsp. *apennina *and *F. gigantea*. Sub-clade A.3 consists of three unresolved samples (*F. mairei*, *F. arundinacea *subsp. *atlantigena *and *F. arundinacea *subsp. *letourneuxiana*) and the two Mediterranean tall fescue varieties. Representatives of the *Drymanthele *sub-genus comprise clade B (99% bootstrap), while clade C (100% bootstrap) contains the fine-leaved species from the *Festuca *sub-genus. *C. cristatus*, *P. pratensis *and *B. distachyon *are out-groups to the entire tree.

#### Acc1

**Figure 3:**
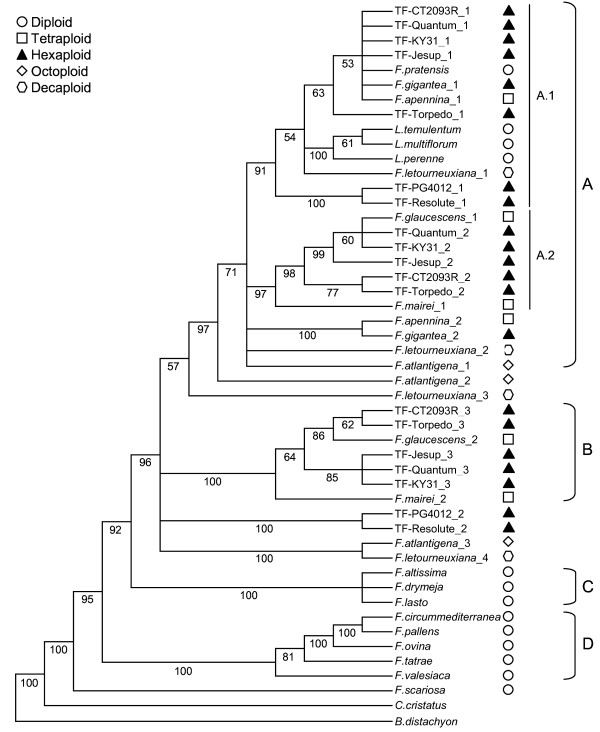
**Strict consensus tree obtained from sequence analysis of the nuclear *Acc1 *gene**. Numbers below branches are bootstrap percentages

The heuristic search conducted on the *Acc1 *data matrix resulted in 504 most parsimonious trees (CI = 0.830; RI = 0.878), the strict consensus of which is shown in Figure 3. This tree can be divided into four major lineages (A-D), in which clades A and B contain representatives of the *Schedonorus*/*Lolium *sub-genus, and clades C and D are assemblages of the diploids from the *Drymanthele *and *Festuca *sub-genera, respectively. Within clade A, haplotypes from *F. arundinacea *subsp. *letourneuxiana *and *F. arundinacea *subsp. *atlantigena*, along with an assemblage of *F. pratensis *subsp. *apennina *and *F. gigantea*, are in sister relationships to the remainder of the clade, which has been further partitioned into sub-clades A.1 and A.2. Sub-clade A.1 (91% of bootstrap) contains haplotypes from the two Mediterranean tall fescue varieties sister to the rest of A.1, which includes the monophyletic *Lolium *species and an unresolved group of one rhizomatous and three Continental tall fescue haplotypes, along with *F. pratensis*, *F. pratensis *subsp. *apennina *and *F. gigantea*. The second rhizomatous tall fescue variety and a haplotype derived from *F. arundinacea *subsp. *letourneuxiana *complete sub-clade A.1. Within sub-clade A.2 (97% of bootstrap), haplotypes from Continental tall fescue are unresolved from an *F. arundinacea *var. *glaucescens *haplotype, while rhizomatous tall fescue haplotypes are sister to this group along with *F. mairei*. *F. arundinacea *subsp. *letourneuxiana *and *F. arundinacea *subsp. *atlantigena *haplotypes are basal to the remainder of clade A. Clade B is well supported (100% of bootstrap) and comprises another group of Continental and rhizomatous tall fescue haplotypes that are related to *F. arundinacea *var. *glaucescens *and again, this group is sister to *F. mairei*. Haplotypes of the two Mediterranean tall fescue samples share a sister relationship, as do the higher-order polyploids (*F. arundinacea *subsp. *atlantigena *and *F. arundinacea *subsp. *letourneuxiana*) and these two groups collapse unresolved alongside clade B. Clade C (100% of bootstrap) is an unresolved monophyletic group consisting solely of the three *Drymanthele *species, while the final clade, D (100% of bootstrap), contains the fine-leaved diploid species of the *Festuca *sub-genus, in which each species is fully resolved. *F. scariosa *holds the most basal position among the *Festuca *species, with *C. cristatus *and *B. distachyon *positioned as out-groups.

#### CEN

**Figure 4:**
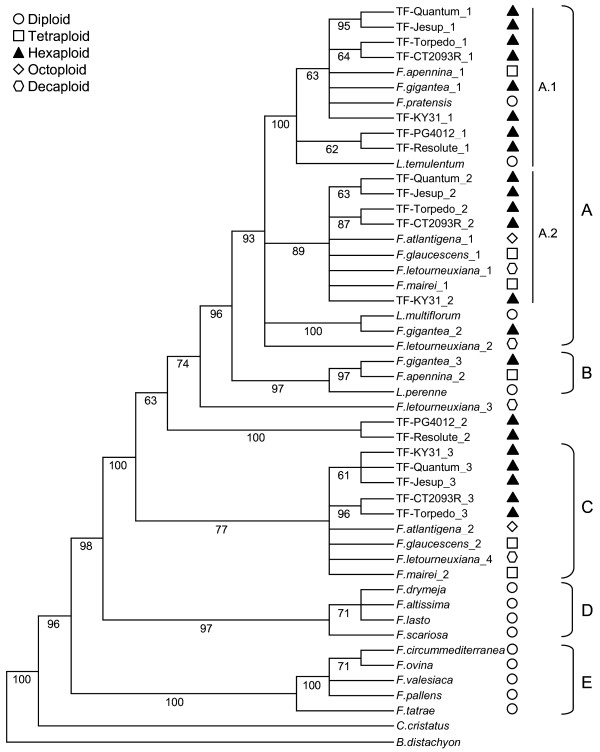
**Strict consensus tree obtained from sequence analysis of the nuclear *CEN *gene**. Numbers below branches are bootstrap percentages

The consensus of 15 most parsimonious trees (CI = 0.852, RI = 0.926) derived from the heuristic search of the *CEN *data matrix is shown in Figure 4. This tree has been divided into five lineages (A-E), in which A, B and C comprise species from the *Schedonorus*/*Lolium *sub-genus, D represents the *Drymanthele *and *Scariosae *sub-genera and E contains species from the fine-leaved *Festuca *sub-genus. Within clade A (93% of bootstrap), the sequence of *L. multiflorum *and haplotypes from *F. gigantea*, *F. arundinacea *subsp. *letourneuxiana *are positioned as sister to two well supported sub-clades (A.1 and A.2). Sub-clade A.1 (100% of bootstrap) is formed from the Continental and rhizomatous tall fescue varieties and unresolved haplotypes from *F. pratensis*, *F. pratensis *subsp. *apennina *and *F. gigantea*. The Mediterranean varieties, along with the *L. temulentum*-derived sequence, then comprise the remainder of A.1. Sub-clade A.2 (89% of bootstrap) again demonstrates close relationships between haplotypes from Continental and rhizomatous tall fescue varieties, and also contains unresolved haplotypes from *F. arundinacea *var. *glaucescens*, *F. mairei*, *F. arundinacea *subsp. *atlantigena*, and *F. arundinacea *subsp. *letourneuxiana*. This monophyletic group is near identical to clade C (77% of bootstrap), which contains haplotypes from all of the same species and displays equivalent structure for *F. arundinacea *var. *glaucescens*, *F. mairei*, *F. arundinacea *subsp. *atlantigena*, and *F. arundinacea *subsp. *letourneuxiana *which are again, all unresolved. The remainder of the *Schedonorus*/*Lolium*-derived haplotypes are either resolved into the small clade B (*F. gigantea*, *F. pratensis *subsp. *apennina *and *L. perenne*: 97% of bootstrap) or are positioned as out-groups to both clades A and B (both Mediterranean varieties and *F. arundinacea *subsp. *letourneuxiana*). The diploids of the *Drymanthele *sub-genus are unresolved within clade D (97% of bootstrap), which also contains the only representative of the *Scariosae *sub-genus (*F. scariosa*), while clade E is a well supported (100% of bootstrap) monophyletic group containing species from the diploid fine-leaved *Festuca *sub-genus. *C. cristatus *and *B. distachyon *form out-groups.

### Congruence between nuclear gene-derived data sets

The *Acc1 *and *CEN *gene-derived phylogenetic trees share similar structure, particularly with respect to relationships between the three tall fescue morphotypes and the division of other *Schedonorus *haplotypes. Both trees reveal comparable evolutionary patterns, *Acc1 *clade A corresponding to *CEN *sub-clade A, and *Acc1 *clade B corresponding to most of *CEN *clade C. The remaining *Acc1 *clades C and D correspond to lineages D and E in the *CEN*-derived dendrogram. The larger source of discordance between the two data sets arises with respect to the *Lolium *species and to *F. scariosa*. While the *Acc1 *gene demonstrates little distinction between the three sampled *Lolium *species, which form a monophyletic group within the *F. pratensis*-containing sub-clade A.1, the *CEN *gene detects a much higher level of variation, such that the three species are distributed throughout the phylogenetic tree in different clades (A.1 - *L. temulentum*, A - *L. multiflorum *and B - *L. perenne*). Similarly, *F. scariosa *exchanges clade location between the two data sets. Within the *Acc1 *gene-derived dendrogram, this species is in a basal position to all other *Festuca*/*Lolium *species, whereas it is positioned as sister to *Drymanthele *sub-genus species in the *CEN *phylogenetic tree. The Incongruence Length Difference test implies significant incongruence between the two nuclear gene data sets (p = 0.01).

## Discussion

### Phylogenetic relationships between each tall fescue morphotype

This phylogenetic study has, for the first time, assessed evolutionary relationships between the three morphotypes of hexaploid tall fescue, which are distinct in geographical and morphophysiological terms. The dendrograms generated from both of the nuclear gene-derived data sets suggest that representatives of the Mediterranean morphotype are genetically distinct from the Continental and rhizomatous varieties sampled here, supporting the observations of hybrid sterility between tall fescue morphotypes [38,39]. It may be assumed that, for each nuclear gene, the three haplotypes recovered from the Continental tall fescue varieties represent the three sub-genomes, as in each case the relevant haplotypes are closely associated with counterparts from *F. pratensis *and *F. arundinacea *var. *glaucescens*. The Mediterranean morphotype-derived nuclear sequences are, however, more distantly related to these progenitor species, implying that they (or closely related taxa) did not participate as direct ancestors of this morphotype. Both the ITS- and *matK *gene-derived dendrograms also support an alternative polyploid origin for the Mediterranean morphotype, despite inability to resolve to the level of individual sub-genomes. For each of these data sets, the Mediterranean varieties fail to be closely associated with either *F. pratensis *or *F. arundinacea *var. *glaucescens*, in contrast to the Continental and rhizomatous samples.

Full interpretation of taxon origin for the polyploid Mediterranean morphotype is rendered difficult due to the recovery of only two distinct sequence haplotypes for each of the nuclear genes. The two Mediterranean variety-derived genotypes may be confidently predicted to be hexaploid in nature, despite a relatively lower estimated genome size as compared to the Continental varietal individuals. This observed difference in genome size was not unexpected, as previous studies have consistently reported lower flow cytometric measurement values from confirmed hexaploid Mediterranean accessions when compared to the samples from the Continental morphotype [79]. Therefore, a relatively trivial explanation for the observation of only two haplotypes is that three distinct sub-genomes are actually present in the Mediterranean morphotype, but one sub-genome is sufficiently diverged from the template primer sequence to be inefficiently amplified and hence under-represented in, or absent from, the contig assemblies. However, the success observed with the *Acc1 *and *CEN*-directed primer pairs across a diverse species range, including more distantly related taxa such as *C. cristatus*, suggests that this interpretation is unlikely. In addition, the depth of sequencing employed proved more than sufficient to recover all three haplotypes from the Continental and rhizomatous tall fescue samples. Alternatively, only two sequence variants may be present in the Mediterranean hexaploids, raising the possibility of an autotetraploid progenitor. In order for disomic inheritance to operate in the contemporary hexaploid, preferential pairing must have arisen between chromosomes of the paired sub-genomes, possibly due to genetic divergence [80], later reinforced by homoeologous pairing gene control [81]. Confirmation of this model would depend on demonstration of haplotype number deficit across a broader selection of nuclear genes, and, ideally, identification of a contemporary autotetraploid taxon related to the putative ancestor. However, no potential progenitors of the Mediterranean morphotype were identified in this study. Such species may be extinct, or have failed to be sampled in taxonomic studies. If still extant, any such progenitors are likely to be taxonomically classified in the *Schedonorus *sub-genus and be located in a similar region (Northern Africa/Western Mediterranean) to both Mediterranean tall fescue and the higher polyploids *F. arundinacea *subsp. *atlantigena *and *F. arundinacea *subsp. *letourneuxiana*. No diploid ancestors of any of the western Mediterranean species (identified by Figure 1 clade A.2) have yet been identified, and so may have become extinct as a result of climate changes associated with glaciation periods, or, less likely, have evaded discovery [1].

The results of this study indicate a close relationship between the Continental and rhizomatous morphotypes, at least for the varieties sampled here, as they are closely associated in both the nuclear gene dendrograms, while displaying sufficient nucleotide variation to be fully resolved from each other. In each instance, the rhizomatous morphotype putative sub-genomic haplotypes are as equally related to *F. pratensis *and *F. arundinacea *var. *glaucescens *as those of Continental individuals, suggesting that these two morphotypes share the same progenitors. The ITS-derived data set provides a distinct indication for separation by a large evolutionary distance, as the Continental and rhizomatous samples are positioned in the 'European' (Clade A.1) and 'Maghrebian' (Clade A.2) sub-clades, respectively. These two sub-clades reflect geographical structure and consistently segregate between the Northern European and African/Western Mediterranean *Schedonorus *species in previous phylogenetic studies performed with ITS sequence [1,4,5]. Information on shared diploid ancestry, however, cannot be obtained through comparison of ITS sequence, as the contributions of each parental genome are generally homogenised to one sequence through the action of either gene conversion or non-homologous unequal crossing-over [82-85]. The evidence of the ITS-derived dendrogram therefore only indicates that the Continental and rhizomatous morphotypes have evolved independently over a sufficient duration to permit differential ITS homogenisation events.

### Phylogenetic inferences from ITS- and *matK*-derived sequence data

The overall general structure of the dendrogram generated from the ITS data set is consistent with those previously published [1,3-6]. As in these studies, the results support the taxonomic classification of Clayton and Renvoize [2] and early divergence of the broad- and fine-leaved *Festuca *species. A lower level of basal resolution was achieved here, however, as the *Schedonorus*, *Festuca *and *Drymanthele *sub-genera form three sister clades (Figure 1, Clades A, B, C) rather than the usual two representing the broad-leaved (*Schedonorus *and *Drymanthele *subgenera) and fine-leaved (*Festuca *sub-genus) species. As clades A and C are less strongly supported (71 and 82% of bootstrap), this disparity is most likely due to minor differences in phylogenetic methodology. In contrast, the *matK *gene was able to effectively resolve the broad and fine-leaved species, and also provided a higher level of resolution in the *Schedonorus *sub-genus (Figure 2, Clade A). Previous phylogenetic analysis of the *Festuca *genus using chloroplast-derived sequence was performed using the *trn*L-*trn*F intergenic spacer [5] and achieved similar resolution to that produced here. The main difference between the ITS- and *matK*-derived dendrograms is the highly supported (92% of bootstrap) formation of sub-clade A.1 (Figure 2) from the *matK *data set, which segregates *F. arundinacea *var. *glaucescens *and both the Continental and rhizomatous tall fescue samples from the remaining 'European' *Schedonorus*/*Lolium *species. As the chloroplast genome is almost always inherited solely from the female parent, this assemblage probably indicates that a taxon closely related to *F. arundinacea *var. *glaucescens *has provided the maternal genome for both the Continental and rhizomatous tall fescue morphotypes. The Mediterranean varieties form a close association with *F. mairei *and the higher polyploids *F. arundinacea *subsp. *atlantigena *and *F. arundinacea *subsp. *letourneuxiana *in the *matK *dendrogram, which raises the possibility of these polyploids sharing a maternal ancestor. While this close relationship with *F. mairei *is not supported by data from the two nuclear genes, it is not feasible to determine whether this is due to the absence of three distinct haplotypes from the Mediterranean varieties or if the association from the chloroplast data is an aberration as a result of low phylogenetic resolution. The *matK *gene analysis, in summary, predicts *F. pratensis *as the maternal genome donor of *F. gigantea *and *F. pratensis *subsp. *apennina*, while one of the G_1 _or G_2 _progenitor genomes appears to have contributed the maternal genome of *F. arundinacea *var. *glaucescens *and both the Continental and rhizomatous tall fescue morphotypes. Mediterranean tall fescue shares a maternal origin with *F. mairei *and the higher polyploids *F. arundinacea *subsp. *atlantigena *and *F. arundinacea *subsp. *letourneuxiana*.

### Possible diploid origins of polyploid *Festuca *species

Although the nuclear gene-derived data sets confirm *F. pratensis *and *F. arundinacea *var. *glaucescens *as probable progenitors of Continental tall fescue, the diploid origins of the G_1 _and G_2 _sub-genomes of *F. arundinacea *var. *glaucescens *could not be determined from either dendrogram. The results do, however, permit some previously proposed diploid species to be excluded as candidate sub-genome donors. *F. altissima *and *F. scariosa *have been previously suggested as diploid progenitors of Continental tall fescue based on morphological comparisons [15] and comparison of chromosome structure [21], but sequences from these two species were never closely associated with any tall fescue haplotype in this phylogenetic analysis. The present results do support cytogenetic studies that indicate an allotetraploid origin for *F. arundinacea *var. *glaucescens *[13], as two distinct haplotypes are observed, and that *F. pratensis *did not contribute either sub-genomes [19]. It has been further proposed that *F. arundinacea *var. *glaucescens *and *F. mairei *may share a diploid sub-genome, based on chromosome structure and the formation of bivalents in hybrids [21,86]. The nuclear gene analysis here indicate a high degree of similarity between both sub-genomes of these tetraploids, to the extent that they are unresolved in the *CEN *gene-derived dendrogram. Sequence from the ITS region was also unable to resolve the two species, and only the chloroplast *matK *gene data set suggests a large evolutionary distance, possibly due to different maternal progenitor taxa.

As the number of recovered nuclear gene haplotypes failed to correspond to the number of expected diploid sub-genomes, resolution of genomic constitutions for the higher polyploids *F. arundinacea *subsp. *atlantigena *(octoploid) and *F. arundinacea *subsp. *letourneuxiana *(decaploid) has not been possible. Nonetheless, the two sub-species are minimally differentiated in all the dendrograms, and contain haplotypes that cannot be resolved from those of *F. arundinacea *var. *glaucescens *and *F. mairei *in the *CEN *gene-derived dendrogram. As the *Acc1 *data set provides higher resolution, the association appears more distant, but it is clear that both *F. arundinacea *var. *glaucescens *and *F. mairei *are closely related to the higher polyploids, and either may have contributed to the formation of these species. Crosses of the two tetraploids produce fertile hybrids that resemble *F. arundinacea *subsp. *atlantigena*, further supporting the speculation that such a hybridisation occurred naturally to produce the octoploid [13]. In this case, the absence of four haplotypes from *F. arundinacea *subsp. *atlantigena *may be due to the inability to discriminate between the *F. arundinacea *var. *glaucescens *and *F. mairei *sub-genome components, which are poorly resolved at the species level by nuclear gene analysis.

All of the phylogenetic trees support strong evolutionary relationships between the 'European' fescue species (*F. pratensis*, *F. pratensis *subsp. *apennina *and *F. gigantea*). Data from both of the nuclear genes suggest that the hexaploid *F. gigantea *was formed through the hybridisation of *F. pratensis *subsp. *apennina *and another diploid species, and that *F. pratensis *is only one of the diploid progenitors of *F. pratensis *subsp. *apennina*. This is consistent with previous studies demonstrating the allopolyploid nature of *F. pratensis *subsp. *apennina *[87] and a common diploid sub-genome (related to *F. pratensis*) between tall fescue and *F. gigantea *[22].

### Phylogenetic utility of *Acc1 *and *CEN *genes

The location of the *B. distachyon Acc1 *and *CEN *orthologues on chromosomes 5 and 4, respectively, is consistent with known macrosynteny between the Triticeae cereals, Poeae grasses and *B. distachyon *[64,65,65,69] and further supports the positioning of these loci on different homoeologous groups within tall fescue. A considerable degree of confidence may therefore be placed in conclusions of hybrid origin, as they are supported by data from two unlinked loci, rather than a single nuclear gene. Both data sets predict very similar relationships between the *Festuca *species, the higher resolution achieved from *Acc1 *being probably due to the result of greater sequence length and the presence of 7 introns (in contrast to 3 in *CEN*), which provide a greater level of nucleotide variation as compared to exons. While the *Acc1 *gene has previously been demonstrated to be effective for clarification of the hybrid origin of polyploid species through phylogenetic reconstruction [59-61], this was the first recorded assessment of *CEN *utility. This gene was originally selected due to verified low copy number in perennial ryegrass [62], along with the presence of introns and ability to be amplified as a single product from the range of species used here. The *CEN *dendrogram was able to confirm the majority of evolutionary relationships observed from the *Acc1 *data set, but the observed polyphyly of *Lolium *species is inconsistent with all other phylogenetic studies, and is possibly an effect of differential selection acting on genes involved in reproductive morphogenesis. Although the multilocus approach used here is crucial for providing independent estimates of evolutionary history, the subsequent independent history of each loci must be considered. *Acc1 *may be described as a 'housekeeping' gene, as it is involved in fatty acid biosynthesis and is hence likely to be subject to strong but even selective pressure across a large range of species. *CEN*, in contrast, is involved in flowering time control, and represses flowering by maintaining the unfixed identity of the inflorescence meristem [62]. Experiments have shown that this gene, among others, is involved in extending the vegetative state of the plant [88-91]. It is therefore possible that *CEN *is under different selective pressure in different species, depending on their geographical distribution and habitat, and that the nucleotide variation reflects these pressures. It is interesting that such variation is only apparent within the *Lolium *species, for which variation in annual-perennial growth habit and floral induction requirements has been well documented. The evolutionary lineages described by *CEN *for the remaining *Festuca *species are the same as those predicted for *Acc1*. In general, the *CEN *gene can be described as useful for phylogenetic reconstruction in the *Festuca*/*Lolium *genera, as it confirmed the majority of relationships produced by *Acc1 *and the ITS data sets. The unexpected placement of *Lolium *species reinforces the importance of comparison between data from multiple unlinked loci when assessing evolutionary relationships.

### Future implications

The results of this study have contributed to the understanding of evolutionary relationships within a group of grass species for which previous taxonomic classifications have been contentious. In particular, a further revision of systematics to recognise the Mediterranean and Continental morphotypes of tall fescue as separate taxa appears to be warranted. The implications of these results, however, reach beyond taxonomy and have the potential to impact on molecular breeding strategies. For example, the demonstration of independent evolution between the Mediterranean and Continental morphotypes suggests sufficient nucleotide variation to allow development of molecular genetic markers capable of discrimination, to enable an uncomplicated germplasm screening method which is simpler and cheaper than sequencing. The ability to distinguish sequences from all three sub-genomes of Continental and rhizomatous tall fescue in this study also has implications for the generation of sub-genome-specific genetic markers, such as single nucleotide polymorphism assays, as has been demonstrated for the outbreeding allotetraploid forage species white clover (*Trifolium repens *L.) [92]. This result implies that the diversity between each sub-genome is sufficient to design sub-genome specific primers, and ultimately detect DNA sequence polymorphisms that are able to attribute homoeologous linkage groups to specific sub-genomes and putative progenitor origins [92,93].

## Conclusions

This study describes the first phylogenetic analysis of the *Festuca *genus to include each of the three tall fescue morphotypes (Continental, Mediterranean and rhizomatous) and has used low copy nuclear gene sequences to identify progenitors of the polyploid species. *F. pratensis *and *F. arundinacea *var. *glaucescens *were confirmed as the probable progenitors of Continental tall fescue, and also as likely ancestors of the rhizomatous morphotype, although these two morphotypes are sufficiently diverse to be positioned in separate clades based on ITS analysis. Phylogenetic reconstruction of all four data sets suggests that Mediterranean tall fescue has evolved independently from both other morphotypes, as a result from hybridisation of different diploid progenitors. These results have implications for taxonomic revision, as well as molecular breeding strategies, and will facilitate the generation of both morphotype and sub-genome-specific molecular genetic markers.

## Authors' contributions

MH carried out the ploidy analysis, extracted the DNA, generated the DNA sequences, performed the phylogenetic analysis and drafted the manuscript. NC and JF co-conceptualised and coordinated the project, contributed to data interpretation and assisted in drafting the manuscript. AS provided plant material, contributed to data interpretation and assisted in drafting the manuscript. All authors read and approved the final manuscript.

## Supplementary Material

Additional file 1 **Relative genome size of each sample**. Genome size was calculated relative to *F. pratensis*, using measurements from both the Ploidy Analyser and previously published estimates. For each sample, Ploidy Analyser measurements represent the average of two readings, with the exception of tall fescue varieties, for which four measurements were made. The previously published estimates represent an average of the respective genome sizes, as reported in multiple prior publications. The extent of error bars represent the standard error of the mean. Click here for file

Additional file 2 **Sequence alignment of the ITS region**. Sequence alignment, in NEXUS format, used to generate the ITS phylogenetic tree Click here for file

Additional file 3 **Sequence alignment of *matK***. Sequence alignment, in NEXUS format, used to generate the *matK *phylogenetic tree Click here for file

Additional file 4 **Sequence alignment of *Acc1***. Sequence alignment, in NEXUS format, used to generate the *Acc1 *phylogenetic tree Click here for file

Additional file 5 **Sequence alignment of *CEN***. Sequence alignment, in NEXUS format, used to generate the *CEN *phylogenetic tree Click here for file

## References

[B1] Inda LA Segarra-Moragues JG Müller J Peterson PM Catalán P Dated historical biogeography of the temperate Loliinae (Poaceae, Pooideae) grasses in the northern and southern hemispheres Molecular Phylogenetics and Evolution 2008 46 3 932 957 10.1016/j.ympev.2007.11.022 18226932

[B2] Clayton W Renvoize S Genera graminum 1986 London, UK: Kew Publishing

[B3] Charmet G Ravel C Balfourier F Phylogenetic analysis in the *Festuca-Lolium *complex using molecular markers and ITS rDNA TAG Theoretical and Applied Genetics 1997 94 8 1038 10.1007/s001220050512

[B4] Torrecilla P Catalán P Phylogeny of broad-leaved and fine-leaved *Festuca *lineages (Poaceae) based on nuclear ITS sequences Systematic Botany 2002 27 2 241 251

[B5] Catalán P Torrecilla P Rodríguez JÁL Olmstead RG Phylogeny of the festucoid grasses of subtribe Loliinae and allies (Poeae, Pooideae) inferred from ITS and *trn*L-F sequences Molecular Phylogenetics and Evolution 2004 31 2 517 541 10.1016/j.ympev.2003.08.025 15062792

[B6] Gaut BS Tredway LP Kubik C Gaut RL Meyer W Phylogenetic relationships and genetic diversity among members of the *Festuca-Lolium *complex (Poaceae) based on ITS sequence data Plant Systematics and Evolution 2000 224 1 33 53 10.1007/BF00985265

[B7] Loureiro J Kopecký D Castro S Santos C Silveira P Flow cytometric and cytogenetic analyses of Iberian Peninsula *Festuca *spp Plant Systematics and Evolution 2007 269 1 89 105 10.1007/s00606-007-0564-8

[B8] Smarda P Bures P Horova L Foggi B Rossi G Genome size and GC content evolution of *Festuca*: ancestral expansion and subsequent reduction Annals of Botany 2008 101 3 421 433 10.1093/aob/mcm307 18158307 PMC2701825

[B9] Terrell EE A taxonomic revision of the genus *Lolium* Technical Bulletin of the United States Department of Agriculture 1968 1392

[B10] Scholz H Ch S Gaisberg MV *Lolium edwardii *sp. nova (Gramineae) and its relationship with *Schedonorus *sect. *Plantynia Dumort* Feddes Repertorium 2000 111 7-8 561 565 10.1002/fedr.20001110722

[B11] Scholz S Scholz H A new species of *Lolium *(Gramineae) from Fuerteventura and Lanzarote (Canary Islands, Spain) Willendowia 2005 35 281 286

[B12] Darbyshire S Realignment of *Festuca *subgenus *Schedonorus *with the genus Lolium (Poaceae) Novon 1993 3 239 243 10.2307/3391460

[B13] Chandrasekharan P Thomas H Studies in *Festuca*. V. Cytogenetic relationships between species of Bovinae and Scariosae Zeitschrift fur Pflanzenzuchtung 1971 65 353 354

[B14] Borrill M Kirby MW Morgan G Studies in *Festuca *11. Interrelationships of some putative diploid ancestor of the polyploid broad-leaved fescues New Phytologist 1977 78 3 661 674 10.1111/j.1469-8137.1977.tb02171.x

[B15] Borrill M Kirby M Morgan G Studies in *Festuca *12. Morphology, distribution and cytogenetics of *F. donax, F*. scariosa and their hybrids, and the evolutionary significance of their fertile amphiploid derivative New Phytologist 1980 86 4 423 439 10.1111/j.1469-8137.1980.tb01683.x

[B16] Malik CP Thomas PT Karyotypic studies in some *Lolium *and *Festuca *species Caryologia 1966 19 167 196

[B17] Malik CP Thomas PT Cytological relationship and genome structure of some *Festuca *species Caryologia 1967 20 1 39

[B18] Seal AG DNA variation in Festuca Heredity 1983 50 3 225 236 10.1038/hdy.1983.26

[B19] Humphreys MW Thomas HM Morgan WG Meredith MR Harper JA Thomas H Zwierzykowski Z Ghesquiere M Discriminating the ancestral progenitors of hexaploid *Festuca arundinacea *using genomic in *situ *hybridization Heredity 1995 75 2 171 174 10.1038/hdy.1995.120

[B20] Pasakinskiene I Anamthawat-Jonsson K Humphreys MW Paplauskiene V Jones RN New molecular evidence on genome relationships and chromosome identification in fescue (*Festuca*) and ryegrass (*Lolium*) Heredity 1998 81 6 659 665 10.1046/j.1365-2540.1998.00446.x

[B21] Harper JA Thomas ID Lovatt JA Thomas HM Physical mapping of rDNA sites in possible diploid progenitors of polyploid *Festuca *species Plant Systematics and Evolution 2004 245 3 163 168 10.1007/s00606-003-0110-2

[B22] Thomas HM Harper JA Meredith MR Morgan WG King IP Physical mapping of ribosomal DNA sites in *Festuca arundinacea *and related species by in situ hybridization Genome 1997 40 406 410 10.1139/g97-054 18464836

[B23] Xu WW Sleper DA Phylogeny of tall fescue and related species using RFLPs *TAG* Theoretical and Applied Genetics 1994 88 6 685 690 10.1007/BF01253971 24186163

[B24] Stammers M Harris J Evans GM Hayward MD Forster JW Use of random PCR (RAPD) technology to analyse phylogenetic relationships in the *Lolium/Festuca *complex Heredity 1995 74 1 19 27 10.1038/hdy.1995.3 7852097

[B25] Reed KFM Clement SL Feely WF Clark B Improving tall fescue (*Festuca arundinacea*) for cool-season vigour Australian Journal of Experimental Agriculture 2004 44 9 873 881 10.1071/EA03173

[B26] Robson M A comparison of British and North African varieties of tall fescue 1. Leaf growth during winter; effects of temperature and day length Journal of Applied Ecology 1967 4 475 484 10.2307/2401349

[B27] Clement SL Elberson LR Youssef NN Davitt CM Doss RP Incidence and diversity of *Neotyphodium *fungal endophytes in tall fescue from Morocco, Tunisia, and Sardinia Crop Science 2001 41 2 570 576 10.2135/cropsci2001.412570x

[B28] Tsai HF Liu JS Staben C Christensen MJ Latch GC Siegel MR Schardl CL Evolutionary diversification of fungal endophytes of tall fescue grass by hybridization with *Epichloe species* Proceedings of the National Academy of Sciences of the United States of America 1994 91 7 2542 2546 10.1073/pnas.91.7.2542 8172623 PMC43405

[B29] Gonzalez-Bernaldez F Borrill M Lindner R Variability of hexaploid Festuca arundinacea 1. Principal components analysis of correlation matrix Boletin de la Real Sociedad Espanola de Historia Natural Seccion Biologica 1969 67 257 263

[B30] Borrill M Tyler BF Lloyd-Jones M Studies in *Festuca *1. A chromosome atlas of Bovinae and Scariosae Cytologia 1971 36 1 14

[B31] Jernstedt JA Bouton JH Anatomy, morphology, and growth of tall fescue rhizomes Crop Science 1985 25 3 539 542 10.2135/cropsci1985.0011183X002500030026x

[B32] Anonymous Torpedo Tall Fescue New Zealand Plant Variety Rights 1994 Grant number FES007

[B33] Stewart AV World First - Tall fescue with rhizomes New Zealand Turf Management Journal 1995 9 32

[B34] Stewart AV The development of a rhizomatous tall fescue (*Festuca arundinacea*) cultivar8th International Turfgrass Research Conference: 1997 1997 Sydney, Australia 136 138

[B35] Charrier S Stewart AV Breeding of rhizomatous turf tall fescue13th Australasian Plant Breeding Conference: 2006 2006 Christchurch, New Zealand 383 l387

[B36] de Bruijin J Tall fescue variety having rhizomes Barenbrug USA, Inc (Tanget, OR) United States 2004 Patent number 6677507

[B37] Ghesquiere M Jadas-Hecart J Prosperi J-Mea Les fe´tuques ou le genre *Festuca* Ressources ge´ne´tiques des plantes fourrage' res et a' gazon 1995 St-Just-La-Pendue, France: BRG

[B38] Hunt KL Sleper DA Fertility of hybrids between two geographic races of tall fescue Crop Science 1981 21 3 400 404 10.2135/cropsci1981.0011183X002100030012x

[B39] Malik CP Thomas PT Chromosomal polymorphism in *Festuca arundinacea* Chromosoma 1966 18 1 1 18 10.1007/BF00326440

[B40] Lewis EJ Hybrids between geographical races of *Festuca arundinacea Schreb* Reports of the Welsh Plant Breeding Station 1963 26 27

[B41] Jadas-Hecart J Gillet M Problems posed by sterile hybrids between two types of tall fescues European and Mediterranean Meeting of the Fodder Crops Section of Eucarpia 1973 1973 Wageningen, the Netherlands

[B42] Drummond JD The effect of hybridisation on seed vigour of Continental and Mediterranean tall fescue (Festuca arundinacea Schreb.) crosses Dissertation Thesis 2006 Lincoln University, New Zealand

[B43] Jauhar PP Tsuchiya T, Gupta PK Recent cytogenetics of the *Festuca-Lolium *complex Chromosome Engineering in Plants: Genetics, Breeding, Evolution 1991 2B Amsterdam, Netherlands: Elsevier Science Publishers 325 362

[B44] Jauhar PP Genetic regulation of diploid-like chromosome pairing in the hexaploid species, *Festuca arundinacea *Schreb. and F. *rubra *L (Gramineae) Chromosoma 1975 52 4 363 382 10.1007/BF00364020

[B45] Jauhar PP Genetic control of diploid-like meiosis in hexaploid tall fescue Nature 1975 254 5501 595 597 10.1038/254595a0 1128654

[B46] Berg CC Webster GT Jauhar PP Buckner RC, Bush LP Cytogenetics and genetics Tall Fescue 1979 Madison, Wisconsin: American Society of Agronomy 93 109

[B47] Eizenga GC Kasperbauer MJ Chromosome pairing in tall fescue haploids derived by anther-panicle culture Journal of Heredity 1985 76 2 99 102

[B48] Soltis PS Soltis DE The role of genetic and genomic attributes in the success of polyploids Proceedings of the National Academy of Sciences of the United States of America 2000 97 13 7051 7057 10.1073/pnas.97.13.7051 10860970 PMC34383

[B49] Sleper DA Janick J Breeding Tall Fescue Plant Breeding Reviews 1985 3 Westport: AVI Publishing Co 313 342

[B50] Sang T Utility of low-copy nuclear gene sequences in plant phylogenetics Critical Reviews in Biochemistry and Molecular Biology 2002 37 3 121 147 10.1080/10409230290771474 12139440

[B51] Fortune PM Schierenbeck KA Ainouche AK Jacquemin J Wendel JF Ainouche ML Evolutionary dynamics of *Waxy *and the origin of hexaploid *Spartina *species (Poaceae) Molecular Phylogenetics and Evolution 2007 43 3 1040 1055 10.1016/j.ympev.2006.11.018 17208463

[B52] Huang S Sirikhachornkit A Su X Faris J Gill B Haselkorn R Gornicki P Genes encoding plastid acetyl-CoA carboxylase and 3-phosphoglycerate kinase of the *Triticum/Aegilops *complex and the evolutionary history of polyploid wheat Proceedings of the National Academy of Sciences of the United States of America 2002 99 12 8133 8138 10.1073/pnas.072223799 12060759 PMC123033

[B53] Vilatersana R Brysting AK Brochmann C Molecular evidence for hybrid origins of the invasive polyploids *Carthamus creticus *and *C. turkestanicus *(Cardueae, Asteraceae) Molecular Phylogenetics and Evolution 2007 44 2 610 621 10.1016/j.ympev.2007.05.008 17591447

[B54] Rousseau-Gueutin M Gaston A Aïnouche A Aïnouche ML Olbricht K Staudt G Richard L Denoyes-Rothan B Tracking the evolutionary history of polyploidy in *Fragaria *L. (strawberry): New insights from phylogenetic analyses of low-copy nuclear genes Molecular Phylogenetics and Evolution 2009 51 3 515 530 10.1016/j.ympev.2008.12.024 19166953

[B55] Small RL Ryburn JA Cronn RC Seelanan T Wendel JF The tortoise and the hare: choosing between noncoding plastome and nuclear *Adh *sequences for phylogeny reconstruction in a recently diverged plant group American Journal of Botany 1998 85 9 1301 1315 10.2307/2446640 21685016

[B56] Gaut BS Morton BR McCaig BC Clegg MT Substitution rate comparisons between grasses and palms: synonymous rate differences at the nuclear gene *Adh *parallel rate differences at the plastid gene *rbcL* Proceedings of the National Academy of Sciences of the United States of America 1996 93 19 10274 10279 10.1073/pnas.93.19.10274 8816790 PMC38374

[B57] Small RL Cronn RC Wendel JF Use of nuclear genes for phylogeny reconstruction in plants Australian Systematic Botany 2004 17 2 145 170 10.1071/SB03015

[B58] Gornicki P Faris J King I Podkowinski J Gill B Haselkorn R Plastid-localized acetyl-CoA carboxylase of bread wheat is encoded by a single gene on each of the three ancestral chromosome sets Proceedings of the National Academy of Sciences of the United States of America 1997 94 25 14179 14184 10.1073/pnas.94.25.14179 9391173 PMC28453

[B59] Huang S Sirikhachornkit A Faris JD Su X Gill BS Haselkorn R Gornicki P Phylogenetic analysis of the acetyl-CoA carboxylase and 3-phosphoglycerate kinase loci in wheat and other grasses Plant Molecular Biology 2002 48 5 805 820 10.1023/A:1014868320552 11999851

[B60] Zhang C Fan X Yu H-Q Zhang H-Q Wang X-L Zhou Y-H Phylogenetic analysis of questionable tetraploid species in *Roegneria *and *Pseudoroegneria *(Poaceae: Triticeae) inferred from a gene encoding plastid acety1-CoA carboxylase Biochemical Systematics and Ecology 2009 37 4 412 420 10.1016/j.bse.2009.04.011

[B61] Fan X Sha L-N Yang R-W Zhang H-Q Kang H-Y Ding C-B Zhang L Zheng Y-L Zhou Y-H Phylogeny and evolutionary history of *Leymus *(Triticeae; Poaceae) based on a single-copy nuclear gene encoding plastid acetyl-CoA carboxylase BMC Evolutionary Biology 2009 9 1 247 10.1186/1471-2148-9-247 19814813 PMC2770499

[B62] Jensen CS Salchert K Nielsen KK A Terminal Flower1-Like Gene from Perennial Ryegrass Involved in Floral Transition and Axillary Meristem Identity Plant Physiol 2001 125 3 1517 1528 10.1104/pp.125.3.1517 11244130 PMC65629

[B63] Cogan N Ponting R Vecchies A Drayton M George J Dracatos P Dobrowolski M Sawbridge T Smith K Spangenberg G Gene-associated single nucleotide polymorphism discovery in perennial ryegrass (*Lolium perenne *L.) Molecular Genetics and Genomics 2006 276 2 101 112 10.1007/s00438-006-0126-8 16708235

[B64] Jones ES Mahoney NL Hayward MD Armstead AP Jones JG Humphreys MO King IP Kishida T Yamada T Balfourier F An enhanced molecular marker based genetic map of perennial ryegrass (*Lolium perenne*) reveals comparative relationships with other Poaceae genomes Genome 2002 45 2 282 295 10.1139/g01-144 11962626

[B65] Sim S Chang T Curley J Warnke SE Barker RE Jung G Chromosomal rearrangements differentiating the ryegrass genome from the Triticeae, oat, and rice genomes using common heterologous RFLP probes TAG Theoretical and Applied Genetics 2005 110 6 1011 1019 10.1007/s00122-004-1916-1 15742203

[B66] Alm V Fang C Busso CS Devos KM Vollan K Grieg Z Rognli OA A linkage map of meadow fescue ( Festuca pratensis Huds.) and comparative mapping with other Poaceae species TAG Theoretical and Applied Genetics 2003 108 1 25 40 10.1007/s00122-003-1399-5 12923626

[B67] Hollingsworth PM Forrest LL Spouge JL Hajibabaei M Ratnasingham S van der Bank M Chase MW Cowan RS Erickson DL Fazekas AJ A DNA barcode for land plants Proceedings of the National Academy of Sciences 2009 106 31 12794 12797 10.1073/pnas.0905845106 PMC2722355 19666622

[B68] Altschul SF Gish W Miller W Myers EW Lipman DJ Basic local alignment search tool Journal of Molecular Biology 1990 215 3 403 410 2231712 10.1016/S0022-2836(05)80360-2

[B69] Initiative TIB Genome sequencing and analysis of the model grass Brachypodium distachyon Nature 2010 463 7282 763 768 10.1038/nature08747 20148030

[B70] Bennett MD Leitch IJ Plant DNA C-values database (release 4.0, Oct. 2005) 2005 http://www.kew.org/cvalues/

[B71] Ceccarelli M Falistocco E Cionini PG Variation of genome size and organization within hexaploid *Festuca arundinacea* TAG Theoretical and Applied Genetics 1992 83 3 273 278 10.1007/BF00224271 24202507

[B72] Hsiao C Jacobs SWL Chatterton NJ Asay KH A molecular phylogeny of the grass family (Poaceae) based on the sequences of nuclear ribosomal DNA (ITS) Australian Systematic Botany 1998 11 6 667 688 10.1071/SB97012

[B73] Tamura K Dudley J Nei M Kumar S MEGA4: Molecular Evolutionary Genetics Analysis (MEGA) Software Version 4.0 Mol Biol Evol 2007 24 8 1596 1599 10.1093/molbev/msm092 17488738

[B74] Rozas J Sanchez-DelBarrio JC Messeguer X Rozas R DnaSP, DNA polymorphism analyses by the coalescent and other methods Bioinformatics 2003 19 18 2496 2497 10.1093/bioinformatics/btg359 14668244

[B75] Young N Healy J GapCoder automates the use of indel characters in phylogenetic analysis BMC Bioinformatics 2003 4 1 6 10.1186/1471-2105-4-6 12689349 PMC153505

[B76] Swofford DL PAUP*. Phylogenetic Analysis Using Parsimony (*and Other Methods) 2003 Version 4.: Sinauer Associates, Sunderland, Massachusetts

[B77] Felenstein J Confidence limits on phylogenies: an approach using the bootstrap Evolution 1985 39 783 791 10.2307/2408678 28561359

[B78] Farris JS Källersjö M Kluge AG Bult C Testing Significance Of Incongruence Cladistics 1994 10 3 315 319 10.1111/j.1096-0031.1994.tb00181.x

[B79] Schardl CL Craven KD Schweri KK Hollin W Clement SL Schmid J West CP Phillips TD Endophytes of the tall fescue ploidy series in Europe, North Africa and the Mediterranean6th International Symposium on Fungal Endophytes of Grasses: 2007 2007 456 Dunedin, New Zealand

[B80] Jenkins G Chatterjee BN Chromosome structure and pairing preferences in autotetraploid rye (Secale cereale) Genome 1994 37 5 784 793 18470122 10.1139/g94-112

[B81] Le Comber SC Ainouche ML Kovarik A Leitch AR Making a functional diploid: from polysomic to disomic inheritance New Phytologist 2010 186 1 113 122 10.1111/j.1469-8137.2009.03117.x 20028473

[B82] Kovarik A Matyasek R Lim KY Skalicka K Koukalova B Knapp S Chase M Leitch AR Concerted evolution of 18-5.8-26S rDNA repeats in Nicotiana allotetraploids Biological Journal of the Linnean Society 2004 82 4 615 625 10.1111/j.1095-8312.2004.00345.x

[B83] Franzke A Mummenhoff K Recent hybrid speciation in *Cardamine *(Brassicaceae) - conversion of nuclear ribosomal ITS sequences in statu nascendi TAG Theoretical and Applied Genetics 1999 98 5 831 834 10.1007/s001220051140

[B84] Rauscher JT Doyle JJ Brown AHD Internal transcribed spacer repeat-specific primers and the analysis of hybridization in the *Glycine tomentella *(Leguminosae) polyploid complex Molecular Ecology 2002 11 12 2691 2702 10.1046/j.1365-294X.2002.01640.x 12453251

[B85] Wendel JF Schnabel A Seelanan T Bidirectional interlocus concerted evolution following allopolyploid speciation in cotton (*Gossypium*) Proceedings of the National Academy of Sciences of the United States of America 1995 92 1 280 284 10.1073/pnas.92.1.280 7816833 PMC42862

[B86] Bowman JG Thomas H Studies in *Festuca*. 8. Cytological relationships between *F. glaucescens *(2n = 28), *F. mairei *(2n = 28) and *F. scariosa *(2n = 14) Zeitschrift fur Pflanzenzuchtung 1976 76 3 250 257

[B87] Clarke J Chandrasekharan P Thomas H Studies in festuca. 9. Cytological studies of Festuca pratensis var. apennina (De Not) Hack. (2n = 28) Zeitschrift fur Pflanzenzuchtung 1976 77 3 205 214

[B88] Fujiwara S Nakagawa M Kamada H Coupland G Mizoguchi T Circadian clock components in Arabidopsis I. The terminal flower 1 enhances the early flowering phenotype of a mutant, lhy cca1 Plant Biotechnology 2005 22 4 311 317

[B89] Szankowski I Waidmann S Omar A Flachowsky H Hattasch C Hanke M RNAi-silencing of MdTFL1 induces early flowering in apple Acta Horticulturae 2009 839 633 636

[B90] Hou C-J Yang C-H Functional Analysis of FT and TFL1 Orthologs from Orchid (Oncidium Gower Ramsey) that Regulate the Vegetative to Reproductive Transition Plant Cell Physiol 2009 50 8 1544 1557 10.1093/pcp/pcp099 19570813

[B91] Foucher F Morin J Courtiade J Cadioux S Ellis N Banfield MJ Rameau C DETERMINATE and LATE FLOWERING Are Two TERMINAL FLOWER1/CENTRORADIALIS Homologs That Control Two Distinct Phases of Flowering Initiation and Development in Pea Plant Cell 2003 15 11 2742 2754 10.1105/tpc.015701 14563931 PMC280576

[B92] Hand M Ponting R Drayton M Lawless K Cogan N Charles Brummer E Sawbridge T Spangenberg G Smith K Forster J Identification of homologous, homoeologous and paralogous sequence variants in an outbreeding allopolyploid species based on comparison with progenitor taxa Molecular Genetics and Genomics 2008 280 4 293 304 10.1007/s00438-008-0365-y 18642031

[B93] Blake NK Sherman JD Dvořák J Talbert LE Genome-specific primer sets for starch biosynthesis genes in wheat TAG Theoretical and Applied Genetics 2004 109 6 1295 1302 10.1007/s00122-004-1743-4 15340684

[B94] Hilu KW Alice LA Liang H Phylogeny of Poaceae inferred from *matK *sequence Annals of the Missouri Botanical Garden 1999 86 4 835 851 10.2307/2666171

[B95] Johnson L Soltis D Phylogenetic inference in Saxifragaceae sensu stricto and *Gilia *(Polemoniaceae) using *matK *sequences Annals of the Missouri Botanical Garden 1995 82 149 175 10.2307/2399875

