# Supplementary figures and images for: Evolutionary history of tall fescue morphotypes inferred from molecular phylogenetics of the Lolium-Festuca species complex
Source: BMC Evol Biol. 2010 Oct 12;10:303. doi: 10.1186/1471-2148-10-303 (PMC2958922; doi:10.1186/1471-2148-10-303)

## Slide 1
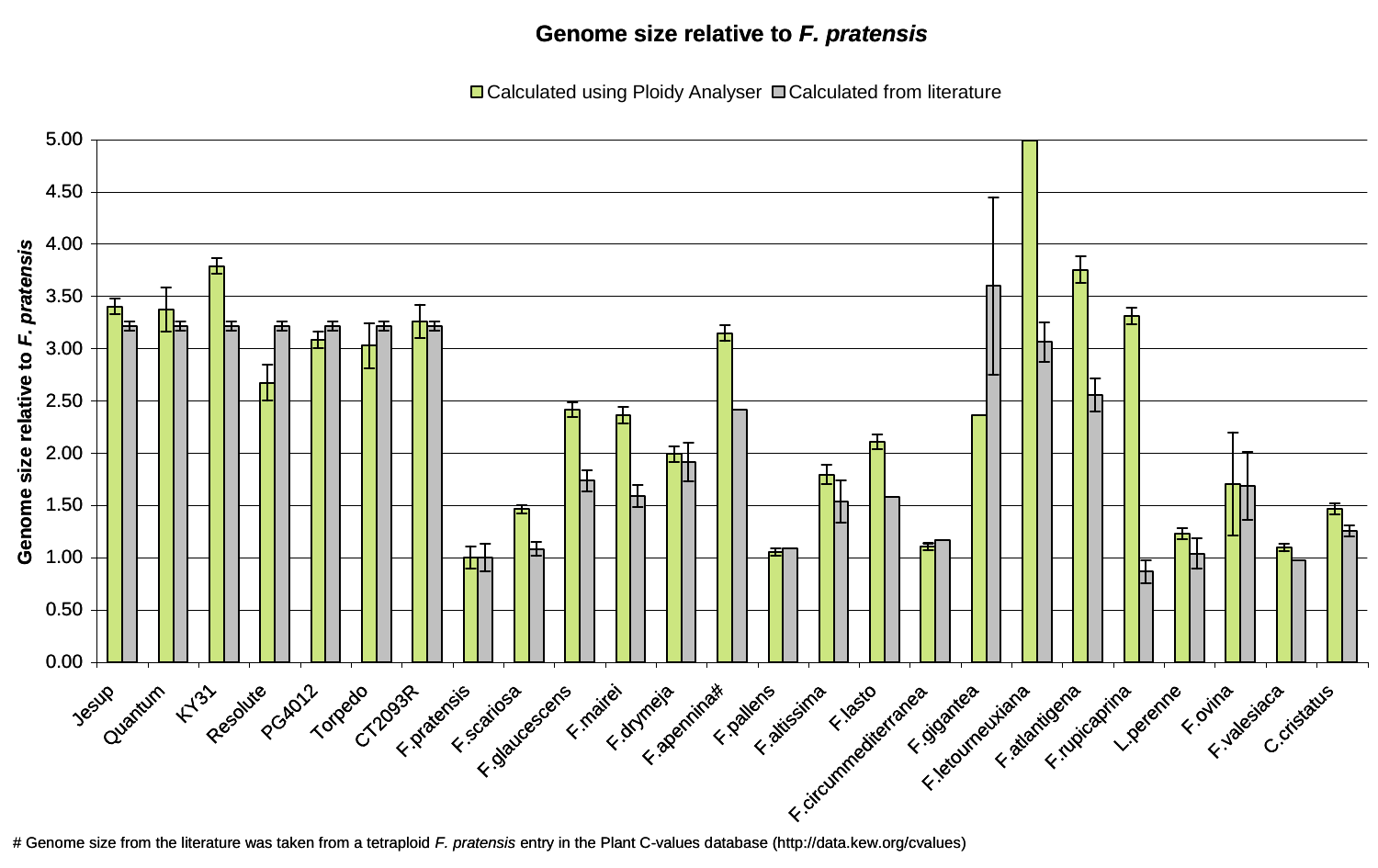

Supplement: Additional file 1 — Relative genome size of each sample. Genome size was calculated relative to F. pratensis, using measurements from both the Ploidy Analyser and previously published estimates. For each sample, Ploidy Analyser measurements represent the average of two readings, with the exception of tall fescue varieties, for which four measurements were made. The previously published estimates represent an average of the respective genome sizes, as reported in multiple prior publications. The extent of error bars represent the standard error of the mean. [file 1471-2148-10-303-S1.PPT]
